# Generating the evidence base for implementation strategies targeting colorectal cancer screening in the accelerating colorectal cancer screening through implementation science (ACCSIS) research projects

**DOI:** 10.1186/s12889-025-26179-2

**Published:** 2026-01-24

**Authors:** Prajakta Adsul, Nidhi Kanabar, Aaron Kruse-Diehr, Mark Dignan, Jill M. Oliveri, Electra D. Paskett, Daniel S. Reuland, Renée M. Ferrari, Alexis A. Moore, Blasé Polite, Karen Kim, Samir Gupta, Sheila F. Castañeda, Maria Elena Martinez, Jennifer Hatcher, Jennifer Coury, Erin S. Kenzie, Amanda F. Petrik, Gloria D. Coronado, David Liebovitz, Jessica Blanchard, Sonja Hoover, Kevin English, Shiraz I. Mishra, Melinda M. Davis, Borsika A. Rabin

**Affiliations:** 1 Division of Epidemiology, Biostatistics, and Preventive Medicine, Department of Internal Medicine, School of Medicine, University of New Mexico, Albuquerque, NM USA; 2 Cancer Control and Population Sciences Research Program, University of New Mexico Comprehensive Cancer Center, Albuquerque, NM USA; 3 Center for Advancing Dissemination and Implementation Science, University of New Mexico, Albuquerque, NM USA; 4 Department of Family and Community Medicine, University of Kentucky College of Medicine, Lexington, KY USA; 5 Department of Medicine, College of Medicine and Comprehensive Cancer Center, The Ohio State University, Columbus, OH USA; 6 Lineberger Comprehensive Cancer Center, University of North Carolina at Chapel Hill, Chapel Hill, NC USA; 7 Department of Maternal and Child Health, Gillings School of Global Public Health, University of North Carolina at Chapel Hill, Chapel Hill, NC USA; 8 Penn State University College of Medicine, Hershey, PA USA; 9 University of Chicago and the Center for Asian Health Equity, Chicago, IL USA; 10 Division of Gastroenterology, Department of Medicine, Moores Cancer Center, University of California San Diego, La Jolla, CA USA; 11 Department of Psychology, San Diego State University, San Diego, CA USA; 12 Herbert Wertheim School of Public Health, Moores Cancer Center, University of California San Diego, La Jolla, CA USA; 13 The University of Arizona Cancer Center, Tucson, AZ USA; 14 Oregon Rural Practice-based Research Network, Oregon Health & Science University, Portland, OR USA; 15 OHSU-PSU School of Public Health, Oregon Health & Science University, Portland, OR USA; 16 Kaiser Permanente Center for Health Research, Portland, OR USA; 17 Department of Medicine, Northwestern University, Chicago, IL USA; 18 Center for Applied Social Research, University of Oklahoma, Norman, OK USA; 19 Implenomics, Dover, DE USA; 20 Albuquerque Area Southwest Tribal Epidemiology Center, Albuquerque Area Indian Health Board, Inc, Albuquerque, NM USA; 21 Departments of Pediatrics and Family and Community Medicine, School of Medicine, University of New Mexico, Albuquerque, NM USA; 22 University of New Mexico Health Sciences Center and its Comprehensive Cancer Center, Albuquerque, NM USA; 23 Department of Family Medicine, Oregon Health & Science University, Portland, OR USA; 24 Herbert Wertheim School of Public Health and Human Longevity Science, University of California San Diego, La Jolla, CA USA; 25 UC San Diego Altman Clinical and Translational Research Institute Dissemination and Implementation Science Center, University of California San Diego, La Jolla, CA USA

**Keywords:** Implementation strategies; colorectal cancer, Screening, Healthcare settings

## Abstract

**Background:**

Implementing evidence-based interventions for population-level benefit can be challenging in resource-limited primary care settings. Research is needed to identify, specify, and systematically study implementation strategies that address the multilevel, contextual influences on implementation in these settings. This study reviewed and compared the implementation strategies proposed by Research Projects (RPs) funded through the Accelerating Colorectal Cancer Screening through Implementation Science (ACCSIS) initiative. ACCSIS research projects implemented multilevel interventions to increase colorectal cancer screening and follow-up among their local populations.

**Methods:**

Participating AC`CSIS RPs provided structured information about activities proposed to facilitate the implementation of evidence-based interventions to increase colorectal cancer screening, follow-up, and referral across project phases (i.e., exploration, preparation, implementation, sustainment). Three implementation science experts reviewed and matched program data to implementation strategies and domains using the Expert Recommendations for Implementing Change (ERIC) classification. ACCSIS RP teams then reviewed and validated matched strategies. Analyses examined similarities and differences among implementation strategies used by each RP and tracked across the screening continuum.

**Results:**

Seven ACCSIS RPs participated in this analysis. Collectively, they reported 89 unique activities that matched 68 ERIC implementation strategies (range: 3 to 17 per site). Several similarities were noted across RPs, such as: four RPs developed and distributed educational materials and three used external facilitation as an implementation strategy. Of the nine domains under which the ERIC strategies are classified, most strategies used by the ACCSIS RPs fell under the domain of using evaluative and iterative strategies (e.g., conducting a local need assessment), followed by training and education (e.g., provider education). All RPs used strategies focused on screening and six used strategies to ensure screening follow-up; only one RP used strategies to improve access to treatment. Most strategies were reported in the preparation and implementation phases.

**Conclusions:**

Systematically documenting and collating implementation strategies across ACCSIS RPs contributes to the evidence base of how multilevel interventions can be implemented to reduce the burden of colorectal cancer through screening and follow-up. Study findings can be used to guide real-world implementation efforts, including future scale-up and sustainment.

**Supplementary Information:**

The online version contains supplementary material available at 10.1186/s12889-025-26179-2.

## Contributions to the literature

- Systematic documentation of implementation strategies in large scale efforts is essential to improving our understanding of what works under what conditions and in which contexts.
- This study is one of the few analyses that systematically documented and reviewed the use of implementation strategies across multiple RPs.
- Findings from this analysis can guide the refinement and contextualization of implementation strategies for colorectal cancer screening.

## Background

The most recent United States Preventive Services Task Force recommendations for colorectal cancer (CRC) screening include fecal testing, flexible sigmoidoscopy, and colonoscopy as effective screening and management interventions to reduce CRC mortality [1]. Expanding the use of proven cancer prevention and early detection interventions is a research priority of the Cancer Moonshot Initiative.^SM^ One Moonshot Initiative, Accelerating Colorectal Cancer Screening through Implementation Science (ACCSIS), aims to build the evidence base for the implementation of multilevel interventions and strategies to increase rates of CRC screening and follow-up to care with a focus on reaching populations for whom screening rates are significantly below national standards. A growing body of work is focused on studying which implementation strategies support the implementation and sustainability of evidence-based interventions (EBIs) across settings and in unique contexts [2, 3]. 

Several multilevel interventions have demonstrated effectiveness in increasing CRC screening uptake and completion for populations experiencing inequities, such as strategies identified by the Guide to Community Preventive Services [4] or published in the National Cancer Institute’s (NCI’s) Evidence-Based Cancer Control Programs [5]. Examples include engaging community health workers or navigators, implementing patient reminders, reducing structural barriers, and using provider reminder and recall systems. A growing consensus is that multilevel interventions—those that target two or more levels of change and address patient, provider, or clinic/organizational/community-level barriers—are needed to achieve national screening targets and reduce persistent screening disparities [6–9]. Despite these strategies, CRC screening and management interventions are often not implemented in routine clinical practice, particularly in settings that traditionally serve low-income, underserved, or historically minoritized communities [10]. Overcoming challenges in implementing EBIs, such as CRC screening for population-level benefit in resource-limited settings, requires a specific focus on implementation strategies. Understanding what strategies should be used and during what implementation phase, and how to best operationalize and evaluate these strategies, are key questions being assessed in the field of implementation science.

Systematic assessments of implementation strategies across studies can allow us to start to answer these questions. Multiple efforts have been initiated to organize and define implementation strategies in the field [11–13]. The Expert Recommendations for Implementing Change (ERIC) compilation has been broadly used in the field and features 73 strategies organized across nine domains, along with associated definitions. Furthermore, there is a great need to better specify what we mean by implementation strategies. Proctor and colleagues provided guidance on how to best name and specify implementation strategies [14]. However, to advance the science of implementation, greater specification is needed to understand how multilevel contextual factors affect the implementation of EBIs and what activities are needed and when to support EBI implementation (i.e., implementation strategies). In this paper, we refer to CRC screening as the EBI. Ultimately, to counter low CRC screening rates, especially in underserved populations, we need an in-depth understanding of the EBI implementation challenges at the patient, provider, and health care system levels and beyond.

Therefore, we conducted a systematic exploration of implementation strategies used by RPs funded through the ACCSIS Moonshot Initiative. Our approach takes advantage of the cross-case comparisons provided by ACCSIS RPs to address four interrelated research questions:

1. In what activities did RPs engage to implement colorectal cancer screening?
2. How did the activities map to ERIC strategies?
3. What were the most common ERIC strategies used overall and during which stage of implementation?
4. Were the strategies used by individual RPs similar or varied?

Our goal for these analyses was to advance pragmatic and conceptual clarity around multilevel context, interventions, implementation strategies, and implementation outcomes using empirical data from ACCSIS RPs. This work helps close critical gaps in the literature and practice by exploring which implementation strategies should be used throughout the stages of implementation (i.e., exploration, preparation, implementation, sustainment) and identifying how to best operationalize and evaluate these strategies for intended impact. Finally, we aimed to understand how multilevel contextual factors affect the implementation of EBIs and what activities are needed and when to support EBI implementation.

## Methods

This was a mixed-methods, observational study conducted as part of the NCI-funded ACCSIS initiative. This analysis was led by members of the Implementation Strategies and Adaptations (ISA) Working Group under the ACCSIS initiative to describe the implementation strategies used in ACCSIS RPs. The workgroup has representation from all eight participating ACCSIS RPs, the coordinating center (Implenomics and RTI International), and NCI. Members of the workgroup have expertise in implementation science, cancer prevention and control research, qualitative and mixed methods, and partnered research with the priority populations engaged in the proposed work. The study received institutional review board approval from University of New Mexico (HRPO# 18–636). Data were collected and analyzed from 2022 to 2024 and included developing the data collection instrument; piloting on three RPs; and collecting data from seven RPs over two rounds of data collection, analyses, and validation.

### Setting and participating ACCSIS RPs

This study was conducted as part of the NCI-funded consortium The Accelerating Colorectal Cancer Screening and Follow-up through Implementation Science (ACCSIS) Program. The overall aim of ACCSIS is to conduct multi-site, coordinated, transdisciplinary research to evaluate and improve colorectal cancer screening processes using implementation science. ACCSIS consists of eight RPs: Oregon Health & Science University/Kaiser Permanente Center for Health Research; University of California, San Diego and San Diego University; University of Arizona Cancer Center; University of Chicago; the University of Kentucky/The Ohio State University; University of New Mexico Comprehensive Cancer Center/Albuquerque Area Southwest Tribal Epidemiology Center; University of North Carolina at Chapel Hill; and University of Oklahoma, Stephenson Cancer Center. The RPs are located across the U.S., and their populations of focus include people in urban, rural, and frontier areas. Three RPs focus specifically on improving CRC uptake among American Indian populations. Seven of the eight ACCSIS RPs participated in the voluntary data collection activities for this study, with one RP deciding that participation in this data collection was beyond their capacity.

### Guiding conceptual frameworks

The data collection activities and analyses were guided by the Exploration, Preparation, Implementation, and Sustainment (EPIS) framework and the ERIC study [12, 15, 16]. We followed the definitions of the implementation phases from the EPIS framework, to guide RPs in documenting the strategies:

- Exploration phase: Evaluate needs and explore potential fit of activities.
- Preparation phase: Planning for implementation of activities.
- Implementation phase: Leadership and support for activities.
- Sustainment phase: Quality assurance.

We were also guided by recommendations from Proctor and colleagues, which informed the set of questions that we asked RPs to help us specify the activities, including the reason why the activity was chosen and what implementation outcomes were being targeted through this activity [14]. 

Implementation strategies are an important component of how to improve health care services. The ERIC study was among the first to advance conceptual clarity around implementation strategies, which are defined as “methods or techniques used to enhance the adoption, implementation, and sustainability of a clinical program or a practice” across multiple studies [14]. The study compiled and defined 73 implementation strategies in the health care literature [12]. As the field has grown around this proposed taxonomy, additional clarity is required for what constitutes implementation strategies in a particular context and how implementation strategies relate to and interact with specific EBI components [17]. 

### Data collection and analyses

Informed by expertise from our workgroup team, review of the ACCSIS RP plans, and our conceptual frameworks (EPIS, ERIC), the study leads (PA, BR, MD) developed a data collection form to gather information from each participating ACCSIS RP regarding key activities they undertook, were currently undertaking, or were planning to undertake to increase the uptake, implementation, and sustained delivery of CRC screening and follow-up within their specific projects (see Appendix 1). We used the following question: “What activities have you undertaken and or are planning to undertake to support the implementation of colorectal cancer screening interventions in clinical settings?” We also requested information about the EBI process phase (i.e., screening, follow-up, referral) and the implementation phase (i.e., exploration, preparation, implementation, or sustainment) of these activities [15, 16]. Finally, we asked which socioecological level(s) (i.e., individual patients, individual providers, community, and health care settings) the activity targeted [18]. Study leads (PA, MD, BAR) of this paper pilot tested and refined the data collection form by applying it to their respective ACCSIS RPs.

Using this refined data synthesis form, we collected primary data from each participating ACCSIS RP by asking the principal investigators and the site representatives from the ISA Work Group to complete the form with input from their clinical implementation partners. After collecting these data, a core group of authors (PA, NK, MD, BAR) undertook a consensus-based review of each activity reported by the ACCSIS RPs and matched them with one primary (except for one occasion) and one to four secondary ERIC strategies based on the descriptions provided by each site. We used the detailed terminology and definitions provided in Appendix 6 of the Powell et al. article to further understand the conceptual basis and operationalization of the 73 ERIC strategies in order to inform the matching process with the activities provided by the RPs [12]. Primary ERIC strategies were considered a direct match for the activity described by the RP. However, we found that using only one primary strategy did not sufficiently capture the complexity or breadth of the activities described by the RPs. Hence, we allowed for the selection of multiple (range:1–4) strategies which were considered and referred to as secondary strategies in this paper.

In addition, we undertook an extensive harmonization process to ensure that strategies and activities matched across the RPs and the strategies were consistently selected and definitions were applied consistently across similar activities. The updated and matched data with the primary and secondary ERIC strategies was returned to each ACCSIS RP for review and confirmation. In this harmonization process, we also noted that RPs often described several activities that matched with the same primary implementation strategy. For the analyses in this paper, we considered these as “unique” implementation strategies within an RP for both primary and secondary strategies.

We also linked the ERIC strategies to the appropriate domain classification, proposed by the conceptual mapping of the ERIC strategies by Waltz and colleagues [19], as follows: (1) Use of evaluative and iterative strategies, (2) Provide interactive assistance, (3) Adapt and tailor to context, (4) Develop stakeholder inter-relationships, (5) Train and educate stakeholders, (6) Support clinicians, (7) Engage consumers, (8) Utilize financial strategies, and (9) Change infrastructure [19]. 

We used iterative, descriptive, mixed-methods to analyze data from individual RPs and across the RPs. We developed summary tables and graphs to represent the number and distribution of primary and secondary strategies reported across ACCSIS RPs. In addition to the summary tables and graphs, we organized the data by the EBI phase (screening, follow-up, referral), implementation phase (EPIS), and socioecological level (individual patients, individual providers, community, and health care setting).

## Results

Results below are organized according to the research questions.

### RQ1. In what activities did RPs engage to implement CRC screening?

Seven ACCSIS RPs provided data for this study. The complete data collected from each RP is in Appendix 2, which includes a brief description of each RP, the activities, the justification, and the related outcomes. Eighty-nine total activities were reported by participating ACCSIS RPs; the number of activities reported by RPs ranged from 3 to 17. These 89 activities mapped onto 90 primary strategies and 134 secondary strategies.

### RQ2. How did the activities map to ERIC strategies?

Table 1 provides the number of activities and the matched primary and secondary ERIC strategies (overall and unique) per RP. After matching the activities, we identified 68 unique ERIC strategies across the participating RPs. Some of these strategies were used multiple times, resulting in 90 primary and 134 secondary ERIC strategies. Because different activities could have been matched to the same ERIC strategy, we also identified the number of unique ERIC strategies specific to each site. The specific details for each activity per RP are in Appendix 2.

**Table 1:** Primary and secondary strategies from the ERIC compilation matched to the activities undertaken by the ACCSIS research projects

Research projects	Activities	Primary	Primary (unique)	Secondary	Secondary (unique)
**RP1**	11	11	7	21	15
**RP2**	3	3	2	4	4
**RP3**	9	9	8	15	10
**RP4**	15	15	13	16	14
**RP5**	19	19	14	31	17
**RP6**	15	15	8	14	7
**RP7**	17	18	16	33	26
**Total**	**89**	**90**	**68**	**134**	**93**

All but one activity was matched to one primary ERIC strategy (Appendix 2). The one activity that was identified with two primary ERIC strategies was “Distribute educational materials such as FAQs that were developed about potential barriers and key questions surrounding care coordination related practices,” which matched with “developing educational materials” and “distributing educational materials.” All activities were assigned at least one secondary ERIC strategy, with some being assigned up to four secondary strategies. For the remaining analyses described in this paper, we used the unique primary strategies (*n* = 68) reported by the seven RPs.

### RQ3. What are the most common strategies used overall and during which stage of implementation?

**Fig. 1:**
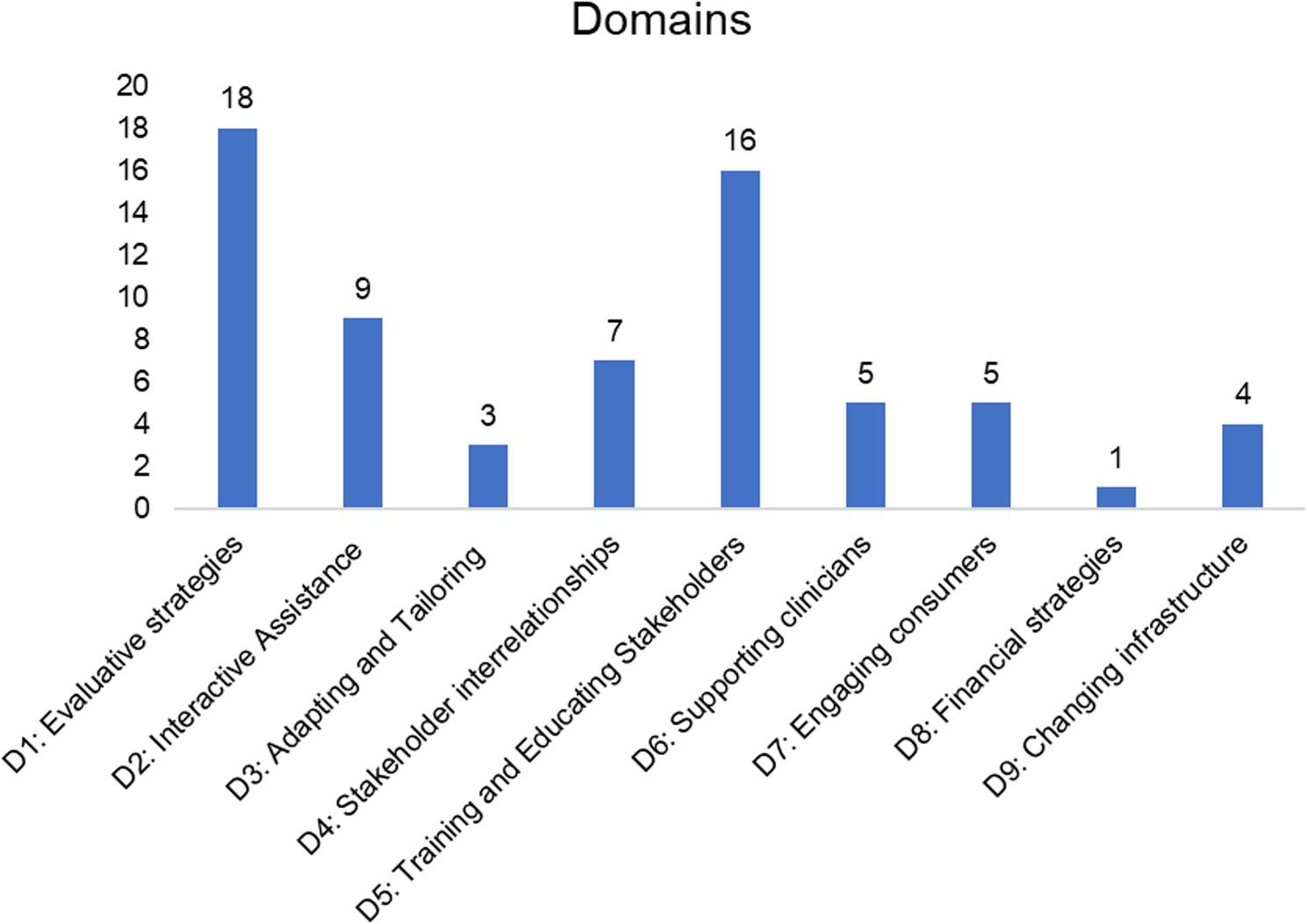
Distribution of matched unique primary strategies (*n*=68) across the nine ERIC strategy domains as specified by Waltz, et. al, 2015. A bar graph showing the distribution of the matched unique primary strategies (*n*=68) across the nine ERIC strategy domains

Figure 1 provides the distribution of unique primary strategies (*n* = 68) across the nine domains of ERIC strategies as specified by Waltz and colleagues [19]. Most matched strategies were from the domains of evaluative or iterative strategies, training and educating stakeholders, and interactive assistance. Of the 73 strategies in the ERIC compilation, activities and strategies described by the RPs aligned with 34 of these strategies. In other words, only 34 of the ERIC strategies were matched on to the activities reported by the participating RPs in this study. Within projects, strategies such as facilitation and organizing and developing quality monitoring systems often mapped onto several activities. The most frequently matched primary strategy was to conduct ongoing training, followed by developing and organizing quality monitoring systems and facilitation.

**Fig. 2:**
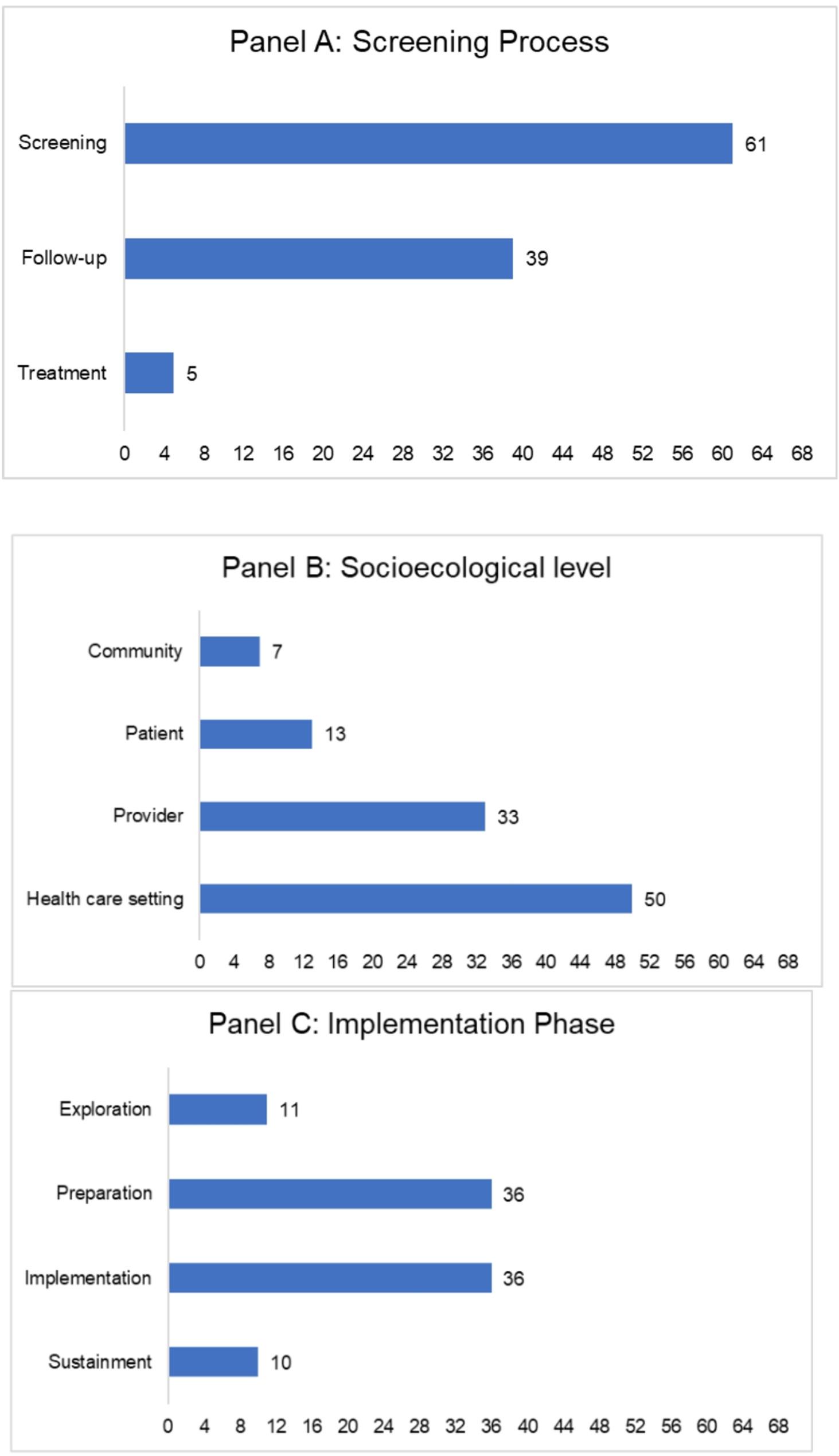
Distribution of unique primary strategies across screening process (Panel **A**), multiple socioecological levels (Panel **B**), and implementation phase (Panel **C**). The figure shows three horizontal bar graphs (panel **A**, **B** and **C**) showing the distribution of the unique primary strategies across screening process (Panel **A**), multiple socioecological levels (Panel **B**), and implementation phase (Panel **C**)

Figure 2 shows the distribution of primary unique strategies (*n* = 68) across the screening and follow-up process, socioecological levels, and the implementation phase. For the screening, follow-up, and referral process (Fig. 2, Panel A), most strategies focused on screening, followed by follow-up and referral. In this case, follow-up included screening positive individuals undergoing diagnostic tests and not just the return of screening tests. With respect to socioecological levels (Fig. 2, Panel B), most strategies targeted the health care setting and providers, with few strategies targeting the community or patients. For the implementation phases (Fig. 2, Panel C), we used the EPIS guidance to define these phases and found most strategies focused on preparation and implementation, with limited focus on exploration and sustainment phases.

### RQ4: were the strategies used by individual RPs similar or varied?

As detailed in Table 2, when examining strategies by participating RPs, the three most-used strategies were conducting ongoing training, developing and organizing quality monitoring systems, and facilitation. All but one site reported conducting ongoing training, making this strategy the most used across RP. Strategies related to adapting or tailoring (Domain 3), changing infrastructure (Domain 9), and financial strategies (Domain 8) were the least used across the projects. Altogether, the RPs used at least one strategy from each of the nine domains.

**Table 2:** Most commonly used primary ERIC strategies by ACCSIS RPs (*N* = 7)

Domain	ERIC Strategy	RP Count
**D5**	Conduct ongoing training	6
**D1**	Develop and organize quality monitoring systems	5
**D2**	Facilitation	4
**D1**	Develop and implement tools for quality monitoring	3
**D1**	Assess readiness and identify barriers and facilitators	3
**D2**	Provide local technical assistance	3
**D3**	Use data warehousing techniques	3
**D5**	Develop educational materials	3
**D5**	Distribute educational materials	3
**D7**	Intervene with patients/consumers to enhance uptake and adherence	3
**D9**	Change record systems	3
**D1**	Conduct local needs assessment	2
**D1**	Audit and provide feedback	2
**D2**	Centralize technical assistance	2
**D3**	Tailor strategies	2
**D4**	Obtain formal commitment	2
**D4**	Identify and prepare champions	2
**D5**	Conduct educational meetings	2
**D6**	Remind clinicians	2
**D7**	Increase demand	2
**D1**	Conduct cyclical small tests of change	1
**D1**	Develop a formal implementation blueprint	1
**D1**	Purposefully re-examine implementation	1
**D4**	Promote network weaving	1
**D4**	Use advisory boards and workgroups	1
**D4**	Build a coalition	1
**D5**	Create learning collaborative	1
**D5**	Provide ongoing consultation	1
**D6**	Create new clinical team	1
**D6**	Develop resource sharing agreements	1
**D6**	Revise professional roles	1
**D8**	Fund and contract for the clinical innovation	1
**D9**	Change service sites	1

D1: Use evaluative and iterative strategies

D2: Provide interactive assistance

D3: Adapt and tailor to context

D4: Develop stakeholder relationships

D5: Train and educate stakeholders

D6: Support clinicians

D7: Engage consumers

D8: Utilize financial strategies

D9: Change infrastructure

## Discussion

Using empirical data, this study was one of the few to systematically collate activities undertaken by RPs within a consortium. Aligning with the ERIC study allowed for the generation of evidence on implementation strategies that could increase the uptake, implementation, and sustainment of CRC screening, follow-up, and referral across seven RPs involved in the ACCSIS Cancer Moonshot^SM^ Initiative. Notably, this initiative is designed to address CRC inequities in primary care settings serving primarily historically minoritized communities. The participating RPs reported 89 activities, which mapped onto 90 primary strategies and 134 secondary strategies. Of these 68 were considered unique primary strategies which aligned with 34 of the ERIC study’s strategies; highlighting the subset of the strategies that may be particularly relevant to CRC screening. Most matched strategies were from the domains of evaluative or iterative strategies, training and educating stakeholders, and interactive assistance. These strategies reflect the project goals of actively assisting implementation of CRC screening interventions in clinical settings. The distribution of the number of strategies and how these were used across the screening continuum, socioecological level, and implementation phase showed considerable variation. Given the focus on screening for all RPs, most strategies focused on screening, followed by follow-up and treatment, most strategies were implemented to support screening in health care settings, and were used in the preparation and implementation phases, depending on the RPs prior research.

Our analysis found that in many cases, several activities were undertaken to accomplish a single ERIC strategy (i.e., activities were coded with both primary and secondary ERIC strategies most of the time). The fact that each program reported a high number of activities and related strategies underlines the complexity of supporting uptake, implementation, and sustained use of EBIs. This finding further suggests that each ERIC strategy may include complex, multistep, and sometimes multilevel activities. Like our study, Gilmartin and colleagues [2] identified many reported strategies when undertaking a postmortem analysis of implementation strategies in their implementation study in the Veterans Health Affairs. Furthermore, many studies might only report a few primary implementation strategies when they design implementation studies but, when prompted, can list several additional secondary strategies or activities that they use to support implementation. These are often not reported when strategies are not collated systematically, but are critical for the success of implementation and reported by our study.

Our analysis of RP activities by implementation phase showed that combinations of strategies vary in different stages of the implementation process and that some strategies may be applicable across all phases [20]. For example, using EPIS as a model, we found that use of evaluative and iterative strategies and developing stakeholder relationships were most reported during the exploration phase, training and education strategies were indicated for implementation, and developing stakeholder relationships and changing infrastructure were selected for sustainment. Notably, we found that few of our projects used strategies specifically focused on sustainment. This may be a limitation because of a lack of focus on sustainment in the ERIC compilation [21] and since many of the projects and partnerships included in this study and funded through the ACCSIS initiative may not have been in the sustainability phase.

In addition, strategies that were applicable across the implementation phases included tailoring strategies, creating learning collaboratives, changing record systems, providing local technical assistance, and using advisory boards and working groups. Thus, guidance is needed not only for what strategies to use in what settings but also for when to use them. Variation in implementation strategy application by study phase is echoed in work by Smith and colleagues [22]. As a note here, although there is guidance from the Centers for Disease Control and Prevention [23] about the term stakeholder, we chose to use it in this paper to align with the strategies as specified in the ERIC study [12, 19]. 

Some RPs reported strategic configurations and combinations of implementation strategies. For example, one RP included three strategies: to obtain formal commitments (D4, 24); develop resource sharing agreements (D6, 49); and fund and contract for the innovation (D8, 57). Another RP included conduct ongoing training (D5, 36), but incorporated an emphasis on making it dynamic (D4, 39) and creating a learning collaborative around it (D5, 44). These examples highlight the importance of combining or integrating strategies to achieve sustainable impact; consistent with the multilevel intervention research agenda [18]. Future research needs to further evaluate how and why these strategies may be more important than discrete, one-time use strategies.

In terms of the operationalization of the implementation strategies using the Proctor criteria, a few areas required additional consideration and therefore harmonization across RPs. First, the activity name did not always match the operationalization of the activity; in these cases, we went with the operationalization, not the activity name (See RP3, A6). Second, the specification of who was undertaking the strategy was not always clearly stated. In some cases, the RPs stated, “We did x,” whereas others specified, “research team did x.” Often missing from the descriptions were considerations around the implementation outcomes and the impact of the strategy. Finally, the justifications for the use of a strategy were very rarely based on theory or previous data (RP6 and RP7 did use theory) and mostly relied on pragmatic justification (i.e., this is what worked before).

Multiple efforts have been initiated to organize and define implementation strategies in the field. The ERIC compilation has been broadly used in the U.S. context and lists 73 strategies organized across nine domains and associated definitions for these strategies. Furthermore, there is a great need to better specify what we mean by implementation strategies. Proctor and colleagues provided guidance on how to best name and specify implementation strategies, but this has yet to be universally adopted. Most recently, a review summarized the evidence to-date for implementation strategies, led by researchers from RAND Health Care in support of the Patient-Centered Outcomes Research Institute (PCORI) [24]. Our study findings map well on to the systematic review in highlighting a high degree of bundling of strategies (as evidenced by 3 to 17 strategies noted per RP), consistent use of education meetings and distributing educational materials across many RPs, and the effectiveness of external facilitation.

A few limitations of this study should be noted. First, we focused on RPs participating in the national ACCSIS initiative and our findings may be specific to interventions addressing CRC. However, we expect that our analytic approach could be applied in other multisite implementation studies. Second, we only asked RPs to report on activities used at the start of this specific research initiative. Thus, we may have missed activities that occurred prior to the research being funded and novel strategies that RPs integrated during the actual implementation period, as noted in prior attempts to synthesize implementation strategies [22]. This dynamic adaptation of activities and implementation strategies may be an important indicator of study team responsiveness to context, rather than a weakness, as it is framed in some adaptation literature [25]. Third, this method of data collection used retrospective data and required considerable effort to harmonize activities and strategies. Specifically, the team needed familiarity with operationalization of ERIC strategies and frequently reviewed the existing definitions to anchor primary and secondary classifications [12]. Although findings may inform research activities, it may be difficult to use this approach in non-research settings. Similar to other methods for tracking implementation strategies, such as the Longitudinal Implementation Strategy Tracking System [26] and other tools [27], our approach was resource-intensive, however, most of the effort was on the research team analyzing the data and not on the implementing partners. Finally, our analysis primarily focused on a single ERIC classification, but most activities fell into multiple ERIC strategies. This is a key opportunity for future research to explore nuances and complexity of strategies, and the form and function of these strategies.

Our study presents opportunities for future research to advance cross-site comparisons, to clarify our classifications for implementation strategies, and to advance methods for the study of implementation strategies. We believe that the synthesis of strategies for health conditions and settings can be critical for practitioners looking for guidance in implementation. Collating strategies across projects can also help create generalizable knowledge, further advancing the science of implementation. Further, our work sets the stage for comparative studies between projects of how variation in coverage of ERIC domains based on the CRC screening and follow up strategies implemented correlate with variation in improvement in these outcomes.

The methodology described and used for this study is novel in a few ways. First, in using Excel-based data collection methods that focused on activities (as opposed to implementation strategies or interventions), we hoped to reduce respondent and implementing partners burden. Second, as a team of implementation scientists, we were able to draw on our extensive expertise to determine the specific strategies that RPs were using, which was important for accuracy and consistency when generating evidence for implementation. Specifically, the analyses were conducted per unique strategy matched with the activities and not the activities themselves, and the analyses were geared toward the primary strategy identified. Finally, this methodology allowed us to capture consistent information across seven RPs that were working toward the same goal.

Some specific recommendations pertaining to the ERIC could be considered in future studies. First, the complexity of electronic health record changes was not well captured in these strategies. Sometimes, all that was needed was an “IT expert to come in and fix something,” whereas other times, “the whole system needed to [be] uprooted.” In some cases, we tried to put this activity under the implementation strategy “change of infrastructure,” but many times, in reading the operationalizations from the RPs, a clear fit could not be established. Furthermore, community engagement and outreach activities were not captured under any of the strategies. Many projects conducted focused activities to adapt educational materials to their relevant context, which is an important strategy but missing from the compilation (it does not quite fit under development of educational materials).

When asked to operationalize the activities, especially to provide justifications, very few projects did so with a focus on equity. For example, strategies such as “changing services sites” were addressing structural barriers of accessing health care, which required considerable resources that may not be reflected in the reductionist approach to identifying and describing these strategies. Furthermore, Domains 8 and 9, which affected financial strategies and infrastructure, were least used in the projects. Future research is needed to carefully examine which strategies RPs used when they were specifically considering sustainability and equity.

The evidence for implementation generated in this study and subsequent studies could guide efforts to design and develop specific implementation strategies to overcome CRC screening, follow-up, and referral to care barriers at multiple levels and facilitate the uptake of evidence-based screening tests for population outcomes. These data provide a global overview of the complexity of implementation of CRC screening in primary health care settings. Future research could explore how well the various activities and strategies contributed to the uptake, implementation, and sustained use of CRC screening interventions and could also explore what factors contributed to RPs selecting certain types of strategies (e.g., expertise, prior experience, etc.).

## Conclusions

This paper reports on one of the few analyses that systematically reviewed the use of implementation strategies across multiple RPs in a research consortium. Findings from this analysis can guide the refinement and contextualization of ERIC strategies for CRC screening and follow-up.

## Supplementary Information

Supplementary Material 1.

Supplementary Material 2.

## Data Availability

All data generated or analyzed during this study are included in this published article and its supplementary information files. Any additional information can be requested from the corresponding author.
