# Supplementary material for: Generating the evidence base for implementation strategies targeting colorectal cancer screening in the accelerating colorectal cancer screening through implementation science (ACCSIS) research projects
Source: BMC Public Health. 2026 Jan 24;26:576. doi: 10.1186/s12889-025-26179-2 (PMC12896049; doi:10.1186/s12889-025-26179-2)
Supplement: Supplementary file 2 — Supplementary Material 2. [file 12889_2025_26179_MOESM2_ESM.pdf]

**Appendix 2. Activities and matched strategies across Research Projects**

| RP | No | Activity | No. | Primary ERIC strategy matched | Domain | Strategy # | No. | Secondary ERIC strategy matched | Domain | Strategy # |
|---|---|---|---|---|---|---|---|---|---|---|
| **RP1.** The Appalachian RP includes six counties in Kentucky and six in Ohio with one FQHC in each county participating in the project. Clinic champions and implementation teams in each clinic choose evidence-based interventions (EBIs) to adapt and implement in their clinic. Interventions are selected based on qualitative data obtained from interviews with patients and providers at the clinic. For clinics that choose to implement patient navigation, a staff member at each clinic is trained by the research team to navigate patients to CRC screening and to follow-up after an abnormal screen. | | | | | | | | | | |
| RP1 | A1 | Research appropriate CRC screening and follow-up evidence-based interventions | RP1.P1 | Provide ongoing consultation | D5 | 37 | RP1.S1 | Conduct local needs assessment | D1 | 7 |
| RP1 | A2 | Research appropriate patient education materials | RP1.P2 | Provide ongoing consultation | D5 | 37 | RP1.S2 | Conduct local needs assessment | D1 | 7 |
| | | | | | | | RP1.S3 | Develop educational materials | D5 | 38 |
| RP1 | A3 | Implementation facilitators collaborate with clinic implementation team and clinic champion(s) to map out CRC screening (in-reach & outreach) and follow-up pathways | RP1.P3 | Develop and organize a quality monitoring system | D1 | 5 | RP1.S4 | Facilitation | D2 | 11 |
| | | | | | | | RP1.S5 | Provide local technical assistance | D2 | 12 |

| | | | | | | | | | | |
|---|---|---|---|---|---|---|---|---|---|---|
| | | | | | | | RP1.S6 | Provide ongoing consultation | D5 | 37 |
| | | | | | | | RP1.S7 | Identify and prepare champions | D4 | 19 |
| | | | | | | | RP1.S8 | Create a learning collaborative | D5 | 44 |
| RP1 | A4 | Meet with clinic implementation team to plan customized, multi-level intervention based on evidence-based interventions (EBIs) | RP1.P4 | Facilitation | D2 | 11 | RP1.S9 | Provide ongoing consultation | D5 | 37 |
| | | | | | | | RP1.S10 | Develop a formal implementation blue print | D1 | 6 |
| RP1 | A5 | Train implementation team in CRC risk assessment for patients | RP1.P5 | Conduct ongoing training | D5 | 36 | RP1.S11 | Promote adaptability | D3 | 16 |
| RP1 | A6 | Host provider education session and intervention kickoff | RP1.P6 | Conduct educational meetings | D5 | 42 | RP1.S12 | Facilitate relay of clinical data to providers | D6 | 47 |
| | | | | | | | RP1.S13 | Organize clinician implementation team meetings | D4 | 20 |
| RP1 | A7 | Train clinic chapion to track data related to CRC screening and follow-up costs | RP1.P7 | Develop and implement tools for quality monitoring | D1 | 4 | RP1.S14 | Conduct ongoing training | D5 | 36 |
| | | | | | | | RP1.S15 | Conduct educational meetings | D5 | 42 |

| | | | | | | | | | |
|---|---|---|---|---|---|---|---|---|---|
| RP1 | A8 | Using clinic EHR to identify patients in need of screening | RP1.P8 | Develop and organize systems for quality monitoring | D1 | 5 | RP1.S16 | Audit and provide feedback | D2 | 2 |
| | | | | | | | RP1.S17 | Conduct local needs assessment | D1 | 7 |
| RP1 | A9 | Offer support and track community CRC screening and promotion events | RP1.P9 | Distributing educational materials | D5 | 40 | RP1.S18 | Intervene with patient/ consumer to enhance uptake | D7 | 53 |
| | | | | | | | RP1. S19 | Use mass media | D7 | 56 |
| RP1 | A10 | Track clinic wide screening and follow-up rates | RP1.P10 | Develop and organize a quality monitoring system | D1 | 5 | RP1.S20 | Audit and provide feedback | D2 | 2 |
| RP1 | A11 | Track county wide screening rates | RP1.P11 | Develop and organize a quality monitoring system | D1 | 5 | RP1.S21 | Audit and provide feedback | D2 | 2 |

**RP2.** The Arizona project, a program of the American Indian (AI) CRC Screening consortium, is one of the three AI-focused projects of ACCSIS. The project uses a hybrid II design to evaluate effectiveness of comprehensive, multilevel/multicomponent EBIs in accordance with recommendations of the Centers for Disease Control and Prevention’s Community Preventive Services Task Force (CPSTF). Currently, five clinics that primarily serve tribal members are participating in the project. The clinics are a combination of Indian Health Service (IHS, Federal or 638; n=3) and FQHCs (n=2). The PNs at each site received training provided by the consortium and additional training, in the form of a full day videoconference or in person meeting conducted by the AZ team. The primary clinical outcome measures are increases in CRC screening rates over baseline and completeness of follow-up and treatment for patients with a positive screening test.

| | | | | | | | | | | |
|---|---|---|---|---|---|---|---|---|---|---|
| RP2 | A1 | Training project-specific facility-based employees to provide patient navigation | RP2.P1 | Conduct ongoing training | D5 | 36 | RP2.S1 | Distribute educational materials | D5 | 38 |
| | | | | | | | RP2.S2 | Involving patient/consumers and family members | D7 | 52 |

| | | | | | | | | | | |
|---|---|---|---|---|---|---|---|---|---|---|
| RP2 | A2 | Group and individual provider academic detailing | RP2.P2 | Conduct ongoing training | D5 | 36 | RP2.S3 | Facilitate relay of clinical data to providers | D6 | 47 |
| RP2 | A3 | Ongoing engagement of IT staff at participating facilities to make EHR modifications | RP2.P3 | Change record systems | D9 | 67 | RP2.S4 | Conduct ongoing training | D5 | 36 |
| **RP3**. The ACCSIS-Chicago research project (RP) aims to implement a multilevel, multicomponent intervention to increase rates of CRC screening, follow-up, and referral-to-care at four FQHCs located in Illinois and Indiana. Findings from this study are used to inform the implementation process of the multilevel intervention in these four FQHCs. Trained PN are housed within each participating FQHC and are embedded into the clinical workflow. PN activities focus on follow up of two cohorts: those who do not complete their FIT testing, and those who do not follow up after an abnormal FIT. The primary outcome is FIT completion and diagnostic colonoscopy follow up. Secondary outcomes include physician, clinic and patient reported experiences. | | | | | | | | | | |
| RP3 | A1 | Conduct pre-implementation organizational readiness assessment | RP3.P1 | Assess readiness and identify barriers and facilitators | D1 | 1 | RP3.S1 | Conduct local needs assessment | D1 | 7 |
| RP3 | A2 | Collect quarterly data on rates of screening order, screening completion, and screening up to date | RP3.P2 | Audit and provide feedback | D1 | 2 | RP3.S2 | Facilitate relay of clinical data to providers | D6 | 47 |
| | | | | | | | RP3.S3 | Using data experts | D3 | 17 |
| | | | | | | | RP3.S4 | Data warehousing techniques | D3 | 18 |
| RP3 | A3 | Centralize data transfer for the patient reminder short message service | RP3.P3 | Develop and organize quality monitoring systems | D1 | 5 | RP3.S5 | Data warehousing techniques | D3 | 18 |
| | | | | | | | RP3.S6 | Use data experts | D3 | 17 |

| | | | | | | | | | | |
|---|---|---|---|---|---|---|---|---|---|---|
| | | | | | | | RP3.S7 | Facilitate relay of clinical data to providers | D6 | 47 |
| | | | | | | | RP3.S8 | Change record systems | D9 | 67 |
| RP3 | A4 | Conduct provider and staff education using academic detailing approach | RP3.P4 | Conduct educational meeting | D5 | 42 | RP3.S9 | Facilitate relay of clinical data to providers | D6 | 47 |
| RP3 | A5 | Conduct in-depth interviews with leadership and focus group sessions with case manager team to understand the current case management workflow and patient navigation challenges | RP3.P5 | Audit and provide feedback | D1 | 2 | RP3.S10 | Assess readiness and identify barriers and facilitators | D1 | 1 |
| | | | | | | | RP3.S11 | Use train-the-trainer strategies | D5 | 41 |
| | | | | | | | RP3.S12 | Conduct ongoing training | D5 | 36 |
| RP3 | A6 | Train case managers to provide CRC specific patient navigation service | RP3.P6 | Conduct ongoing training | D5 | 36 | RP3.S13 | Intervene with patients/consumers to enhance uptake and adherence | D7 | 53 |
| RP3 | A7 | Use postcards to introduce the short message service to patients | RP3.P7 | Intervene with patients/consumers to enhance uptake and adherence | D7 | 53 | | | | |

| | | | | | | | | | | |
|---|---|---|---|---|---|---|---|---|---|---|
| RP3 | A8 | Work with partner health system to conduct community outreach activities in their catchment areas | RP3.P8 | Increase demand | D7 | 55 | RP3.S14 | Intervene with patients/consumers to enhance uptake and adherence | D7 | 53 |
| RP3 | A9 | Develop staff CRC resource guide and patient CRC resource guide (digital and print versions) | RP3.P9 | Develop educational materials | D5 | 38 | RP3.S15 | Distribute educational materials | D5 | 40 |
| **RP4.** The New Mexico research project, a program of the AI CRC Screening consortium, is one of the three AI-focused projects of ACCSIS. The project uses a hybrid II design to evaluate effectiveness of comprehensive, multilevel/multicomponent EBIs in accordance with recommendations of the CPSTF. Four rurally situated Tribes that operate their own healthcare facilities are participating in the project. Multisector action teams, established and mobilized at the healthcare facilities identify and prioritize the selected EBIs. The teams have elected to focus on community- and system-level multicomponent interventions. The project provided training for PNs recruited by all three AI projects. The primary clinical outcome measures are increases in CRC screening rates over baseline and completeness of follow-up and treatment for patients with an abnormal screening test result. | | | | | | | | | | |
| RP4 | A1 | Improve and update Electronic Health Records (EHR) at clinical site | RP4.P1 | Provide local technical assistance | D2 | 12 | RP4.S1 | Change record system | D9 | 67 |
| RP4 | A2 | Identifying and supporting a clinical champion | RP4.P2 | Identify and prepare champions | D4 | 19 | RP4.S2 | Facilitation | D2 | 11 |
| RP4 | A3 | Creating multi-sector action teams (MAT) at the health clinic | RP4.P3 | Create new clinical team | D6 | 51 | RP4.S3 | Recruit, designate, and train for leadership | D4 | 21 |
| RP4 | A4 | Train patient navigators at the clinical sites | RP4.P4 | Conduct ongoing training | D5 | 36 | RP4.S4 | Making training dynamic | D5 | 39 |
| | | | | | | | RP4.S5 | Creating a learning collaborative | D5 | 44 |

| | | | | | | | | | |
|---|---|---|---|---|---|---|---|---|---|
| RP4 | A5 | Facilitating regular check-ins with multi-sector action teams | RP4.P5 | Facilitation | D2 | 11 | RP4.S6 | Conduct local consensus discussions | D4 | 26 |
| RP4 | A6 | Actively monitoring screening rates and goal setting | RP4.P6 | Develop and organize quality monitoring systems | D1 | 5 | RP4.S7 | Facilitate relay of clinical data to providers | D6 | 47 |
| RP4 | A7 | Assessment and feedback of providers performance | RP4.P7 | Audit and provide feedback | D1 | 2 | RP4.S8 | Facilitate relay of clinical data to providers | D6 | 47 |
| RP4 | A8 | Provider detailing | RP4.P8 | Conduct ongoing training | D5 | 36 | RP4.S9 | Develop educational materials | D5 | 38 |
| RP4 | A9 | Provider reminders | RP4.P9 | Remind clinicians | D6 | 48 | RP4.S10 | Change record system | D9 | 67 |
| RP4 | A10 | Adapting patient educational materials | RP4.P10 | Tailor strategies | D3 | 15 | RP4.S11 | Promote adaptability | D3 | 16 |
| RP4 | A11 | Sending reminders to patients for screening appointments | RP4.P11 | Intervene with patients/ consumers to enhance uptake and adherence | D7 | 53 | RP4.S12 | Develop and organize quality monitoring systems | D1 | 5 |
| RP4 | A12 | Providing patients incentive to return FIT tests | RP4.P12 | Intervene with patients/ consumers to enhance uptake and adherence | D7 | 53 | RP4.S13 | Alter incentive/ allowance structures | D8 | 60 |
| RP4 | A13 | Distributing patient educational materials | RP4.P13 | Distribute educational materials | D5 | 40 | RP4.S14 | Increase demand | D7 | 55 |
| RP4 | A14 | Educating patients through community-bsed education | RP4.P14 | Increase demand | D7 | 55 | RP4.S15 | Distribute educational materials | D5 | 40 |

| | | | | | | | | | | |
|---|---|---|---|---|---|---|---|---|---|---|
| RP4 | A15 | Disseminate FIT tests at community events | RP4.P15 | Change service sites | D9 | 70 | RP4.S16 | Intervene with patients/ consumers to enhance uptake and adherence | D7 | 53 |
| **RP5.** The ACCSIS-North Carolina RP is a hybrid trial type 2, pragmatic randomized controlled trial of centralized mailed FIT outreach and patient navigation in two FQHCs in North Carolina, encompassing 17 clinics and serving ~22,000 age-eligible patients (ages 50–75). The multi-level intervention includes a screening registry of average-risk patients due for CRC screening; mailed FIT outreach to registry patients; and navigation support for colonoscopy completion for patients with an abnormal FIT result. The centralized, trained PN has access to the EHR record at the FQHC and uses phone-based navigation and protocols adapted from Butterly.44 Financial assistance is provided to uninsured patients. | | | | | | | | | | |
| RP5 | A1 | Conduct local needs assessment | RP5.P1 | Conduct local needs assessment | D1 | 7 | | | | |
| RP5 | A2 | Conduct local consensus discussions | RP5.P2 | Conduct local consensus discussion | D4 | 26 | RP5.S1 | Conduct local needs assessment | D1 | 7 |
| RP5 | A3 | Build a coalition | RP5.P3 | Build a coalition | D4 | 23 | RP5.S2 | Visit other sites | D4 | 31 |
| | | | | | | | RP5.S3 | Capture and share local knowledge | D4 | 28 |
| | | | | | | | RP5.S4 | Promote network weaving | D4 | 35 |
| | | | | | | | RP5.S5 | Develop academic partnerships | D4 | 34 |
| RP5 | A4 | Assess readiness and identify barriers and facilitators | RP5.P4 | Assess readiness and identify barriers and facilitators | D1 | 1 | RP5.S6 | Identify and prepare champions | D4 | 19 |
| RP5 | A5 | Use advisory boards and workgroups | RP5.P5 | Use advisory boards and workgroups | D4 | 28 | RP5.S7 | Organize clinician implementation team meetings | D4 | 20 |
| RP5 | A6 | Re-examine implementation | RP5.P6 | Purposefully re-examine implementation | D1 | 3 | RP5.S8 | Assess readiness and identify barriers and facilitators | D1 | 3 |
| | | | | | | | RP5.S9 | Provide local technical assistance | D2 | 12 |

| | | | | | | | | | | |
|---|---|---|---|---|---|---|---|---|---|---|
| | | | | | | | RP5.S10 | Tailor strategies | D3 | 15 |
| RP5 | A7 | Develop a formal implementation blueprint | RP5.P7 | Develop a formal implementation blueprint | D1 | 6 | RP5.S11 | Promote adaptability | D3 | 16 |
| | | | | | | | RP5.S12 | Conduct cyclical small tests of change | D1 | 10 |
| RP5 | A8 | Develop educational materials for patients for screening and follow-up | RP5.P8 | Develop educational materials | D5 | 38 | RP5.S13 | Distribute educational materials | D5 | 40 |
| RP5 | A9 | Develop educational materials for CHC stakeholders (providers, frontline clinic staff) around screening and follow-up | RP5.P9 | Develop educational materials | D5 | 38 | RP5.S14 | Distribute educational materials | D5 | 40 |
| RP5 | A10 | Use data warehousing techniques (i.e., Integrate EHR data from multiple sources to develop a centralized CRC screening registry). | RP5.P10 | Use data warehousing techniques | D3 | 18 | RP5.S15 | Provide local technical assistance | D2 | 12 |
| RP5 | A11 | Conduct cyclical small tests of change | RP5.P11 | Conduct cyclical small tests of change | D1 | 10 | RP5.S16 | Conduct local needs assessment | D1 | 7 |
| | | | | | | | RP5.S17 | Develop and implement tools for quality monitoring | D1 | 4 |

| | | | | | | | | | | |
|---|---|---|---|---|---|---|---|---|---|---|
| RP5 | A12 | Obtain formal commitments | RP5.P12 | Obtain formal commitment | D4 | 24 | RP5.S18 | Develop academic partnerships | D4 | 34 |
| | | | | | | | RP5.S19 | Promote network weaving | D4 | 35 |
| RP5 | A13 | Develop resource sharing agreements | RP5.P13 | Develop resource sharing agreements | D6 | 49 | RP5.S20 | Develop academic partnerships | D4 | 34 |
| RP5 | A14 | Fund and contract for the clinical innovation | RP5.P14 | Fund and contract for the clinical innovation | D8 | 57 | RP5.S21 | Obtain formal commitment | D4 | 24 |
| RP5 | A15 | Centralize the intervention | RP5.P15 | Change record system | D9 | 67 | RP5.S22 | Centralize technical assistance | D2 | 14 |
| | | | | | | | RP5.S23 | Develop and implement tools for quality monitoring | D1 | 4 |
| | | | | | | | RP5.S24 | Develop and organize quality monitoring systems | D1 | 5 |
| RP5 | A16 | Use data warehousing techniques (i.e., Integrate EHR data from multiple sources to develop a centralized CRC screening registry). | RP5.P16 | Use data warehousing techniques | D3 | 18 | RP5.S25 | Provide local technical assistance | D2 | 12 |
| RP5 | A17 | Conduct cyclical small tests of change | RP5.P17 | Conduct cyclical small tests of change | D1 | 10 | RP5.S26 | Conduct local needs assessment | D1 | 7 |
| | | | | | | | RP5.S27 | Develop and implement tools for quality monitoring | D1 | 4 |
| | | | | | | | RP5.S28 | Tailor strategies | D3 | 15 |
| RP5 | A18 | Asess costs and cost effectiveness | RP5.P18 | Develop and organize quality monitoring systems | D1 | 5 | | | | |

| | | | | | | | | | | |
|---|---|---|---|---|---|---|---|---|---|---|
| RP5 | A19 | Support centralized intervention | RP5.P19 | Change record system | D9 | 67 | RP5.S29 | Centralize technical assistance | D2 | 14 |
| | | | | | | | RP5.S30 | Develop and implement tools for quality monitoring | D1 | 4 |
| | | | | | | | RP5.S31 | Develop and organize quality monitoring systems | D1 | 5 |
| **RP6.** The Oregon ACCSIS research project (RP) is a parallel, two-group, cluster-randomized trial of mailed FIT outreach and patient navigation involving Medicaid enrollees who receive primary care at any of 29 participating rural clinic units, which consist of individual or groups of clinics (n = 15 intervention units and n = 14 control units). These clinics are clustered within regional Medicaid health plans, which collaborate on intervention support and implementation. Patient navigators (PNs) are diverse clinic employees (e.g., medical assistants, Community Health Workers) with a back-up PNs provided at the health plan level. PN activities are focused on navigating to follow-up colonoscopy for enrollees with an abnormal stool test result. Clinic staff serving as PNs from each practice are trained in an adapted version of the New Hampshire patient navigation program.44 Training consists of 6 hours of pre-recorded videos and two 90-minute interactive sessions; implementation facilitation and booster training is offered during monthly group meetings among research staff, clinic staff (including PNs), and Medicaid health plan staff. Primary effectiveness outcome is completion of colonoscopy within 6 months of an abnormal fecal test result. | | | | | | | | | | |
| RP6 | A1 | Research team provided instruction to clinics to identify population | RP6.P1 | Provide local technical assistance | D2 | 12 | RP6.S1 | Develop and organize quality monitoring systems | D1 | 5 |
| RP6 | A2 | Clinics identify population due for screening | RP6.P2 | Develop and implement tools for quality monitoring | D1 | 4 | RP6.S2 | Develop and organize quality monitoring systems | D1 | 5 |
| RP6 | A3 | Research team encouraged and reminded CCOs to pull list according to data pull requirements | RP6.P3 | Provide local technical assistance | D2 | 12 | RP6.S3 | Develop and organize quality monitoring systems | D1 | 5 |
| RP6 | A4 | Research team checked the data for inconsistencies from CCO prior to | RP6.P4 | Provide local technical assistance | D2 | 12 | RP6.S4 | Use data warehousing techniques | D3 | 18 |

| | | | | | | | | | | |
|---|---|---|---|---|---|---|---|---|---|---|
| | | sharing with clinics | | | | | | | | |
| RP6 | A5 | Clinics scrub the CCO/research team QCed list of patients for eligibility for mailed FIT | RP6.P5 | Develop and organize quality monitoring systems | D1 | 5 | RP6.S5 | Use data warehousing techniques | D3 | 18 |
| RP6 | A6 | Training for clinic staff in mailed FIT and clinic-level chart scrubbing | RP6.P6 | Conduct ongoing training | D5 | 36 | RP6.S6 | Provide ongoing consultation | D5 | 37 |
| RP6 | A7 | Practice facilitators conducted workflow assessments with clinics for mailed FIT and navigation workflows | RP6.P7 | Facilitation | D2 | 11 | RP6.S7 | Purposefully re-examine the implementation | D1 | 3 |
| | | | | | | | RP6.S8 | Provide local technical assistance | D2 | 12 |
| RP6 | A8 | Facilitation with CCOs to select a mailing vendor and order FITs, set up postage requirements | RP6.P8 | Facilitation | D2 | 11 | RP6.S9 | Provide local technical assistance | D2 | 12 |
| RP6 | A9 | Prompt letters, phone calls, and/or texts to patients due for CRC screening, before the FIT mailing | RP6.P9 | Intervene with patients/consumers to enhance uptake and adherence | D7 | 53 | | | | |

| | | | | | | | | | | |
|---|---|---|---|---|---|---|---|---|---|---|
| RP6 | A10 | CCOs mailed FIT kits to the list of patients (verified by clinic review) | RP6.P10 | Centralize technical assistance | D2 | 14 | RP6.S10 | Change service sites | D9 | 70 |
| RP6 | A11 | CCOs and clinics sent patient reminder texts, letters, and phone calls to return the FITs | RP6.P11 | Intervene with patients/consumers to enhance uptake and adherence | D7 | 53 | | | | |
| RP6 | A12 | Facilitators provided support to clinics and CCOs for reminders | RP6.P12 | Facilitation | D2 | 11 | RP6.S11 | Provide local technical assistance | D2 | 12 |
| RP6 | A13 | Patient Navigators (identified during workflow assessments) are trained in navigation to follow-up colonoscopy | RP6.P13 | Revise professional roles | D6 | 50 | RP6.S12 | Conduct ongoing training | D5 | 36 |
| RP6 | A14 | Patients with abnormal FITs are navigated to follow-up colonoscopy | RP6.P14 | Intervene with patients/consumers to enhance uptake and adherence | D7 | 53 | | | | |
| RP6 | A15 | Provide ongoing facilitation support to clinics and CCOs through FIT mailing and navigation activities | RP6.P15 | Facilitation | D2 | 11 | RP6.S13 | Provide local technical assistance | D2 | 12 |
| | | | | | | | RP6.S14 | Provide ongoing consultation | D5 | 37 |

| | | | | | | | | | | |
|---|---|---|---|---|---|---|---|---|---|---|
| **RP7.** The San Diego ACCSIS RP is a pragmatic cluster-randomized trial to assess whether a Hub-and-Spoke multilevel intervention increases CRC screening through mailed FIT outreach and abnormal FIT follow-up through patient navigation. The base population is three community health center systems that oversee 7 to 9 clinics each, totaling 25 clinics. The patient navigation program includes telephone-based care coordination for colonoscopy scheduling, preparation, and follow-up. PNs are federally qualified health center (FQHC) employees who are trained in the ACCSIS San Diego patient navigation program; program materials include abnormal FIT follow-up key activities description, data tracking logs for intervention activities and outcomes, barriers and solutions addressed, referrals and frequently asked questions. Effectiveness of abnormal FIT follow-up patient navigation will be measured as the proportion with abnormal FIT who complete diagnostic colonoscopy within 6 months. | | | | | | | | | | |
| RP7 | A1 | Care coordination training | RP7.P1 | Conduct ongoing training | D5 | 36 | RP7.S1 | Conduct educational meetings | D5 | 45 |
| | | | | | | | RP7.S2 | Develop educational materials | D5 | 38 |
| | | | | | | | RP7.S3 | Distribute educational materials | D5 | 40 |
| | | | | | | | RP7.S4 | Facilitation | D2 | 11 |
| | | | | | | | RP7.S5 | Local technical assistance | D2 | 12 |
| | | | | | | | RP7.S6 | Promote network weaving | D4 | 35 |
| RP7 | A2 | Distribute educational materials such as FAQs about potential barriers and key questions surrounding care coorindation related practices | RP7.P2 | Develop educational materials | D5 | 38 | RP7.S7 | Assess readiness and identify barriers and facilitators | D1 | 1 |
| | | | RP7.P3 | Distribute educational materials | D5 | 40 | | | | |
| RP7 | A3 | Brainwriting excersise | RP7.P4 | Assess readiness and identify barriers and facilitators | D1 | 1 | RP7.S8 | Audit and provide feedback | D1 | 2 |

| | | | | | | | | | | |
|---|---|---|---|---|---|---|---|---|---|---|
| | | | | | | | RP7.S9 | Capture and share local knowledge | D4 | 27 |
| RP7 | A4 | Assessing context and group action planning/individual and group-based assessment of context and fit | RP7.P5 | Assess readiness and identify barriers and facilitators | D1 | 1 | RP7.S10 | Purposefully reexamine implementation | D1 | 3 |
| | | | | | | | RP7.S11 | Promote adaptability | D3 | 16 |
| RP7 | A5 | Working with the Hub partner (HQP) to coordinate mailed FIT outreach, Care Coordination, and additional study related activities | RP7.P6 | Promote network weaving | D4 | 35 | RP7.S12 | Organize clinician implementation team meetings | D4 | 20 |
| | | | | | | | RP7.S13 | Centralize technical assistance | D2 | 14 |
| | | | | | | | RP7.S14 | Create a learning collaborative | D5 | 44 |
| RP7 | A6 | Building bidirectional relationships using multi-stakeholder meetings with health center partners (ongoing meetings w/ CHC and the Hub partner/ongoing technical assistance) | RP7.P7 | Provide local technical assistance | D2 | 12 | RP7.S15 | Promote network weaving | D4 | 35 |
| RP7 | A7 | Use of a centralized | RP7.P8 | Change record systems | D9 | 67 | RP7.S16 | Develop and implement tools for quality monitoring | D1 | 4 |

| | | | | | | | | | | |
|---|---|---|---|---|---|---|---|---|---|---|
| | | (Arcadia) HER for all health centers | | | | | | | | |
| RP7 | A8 | Assessments-fidelity assessment to assess QI | RP7.P9 | Develop and implement tools for quality monitoring | D1 | 4 | RP7.S17 | Develop and organize quality monitoring system | D1 | 5 |
| RP7 | A9 | Sub-contract agreements and Buisness Associate Agreements between grantee and Hub partner. Subcontracts between the Hub and Spokes (CHCs). BAAs between the CHCs and 3rd party vendor (Prevlon) BAA/Contract between the Hub and 3rd party vendor | RP7.P10 | Obtain formal commitments | D4 | 24 | RP7.S18 | Develop resource sharing agreements | D6 | 49 |
| | | | | | | | RP7.S19 | Fund and contract for the innovation | D8 | 57 |
| RP7 | A10 | Activate champions and partners and identify innovators (early adopters) through decade long relationship building and initial invitations | RP7.P11 | Identify and prepare champions | D4 | 19 | RP7.S20 | Identify early adopters | D4 | 25 |

| | | | | | | | | | | |
|---|---|---|---|---|---|---|---|---|---|---|
| RP7 | A11 | Annual Colorectal Cancer Roundtable - community health center focused annual event- engaging informal opinion leaders, etc | RP7.P12 | Create learning collaborative | D5 | 44 | RP7.S21 | Promote network weaving | D4 | 35 |
| | | | | | | | RP7.S22 | Build a coalition | D4 | 34 |
| | | | | | | | RP7.S23 | Inform local opinion leaders | D4 | 22 |
| | | | | | | | RP7.S24 | Capture and share local knowledge | D4 | 27 |
| RP7 | A12 | Implementation facilitation team (help partner)/ centralize technical assistance | RP7.P13 | Centralize technical assistance | D2 | 14 | | | | |
| RP7 | A13 | Care cordinators function (example remind providers to adress all processes of the care cordination activities) | RP7.P14 | Remind clinicians | D6 | 48 | RP7.S25 | Facilitate relay of data to clinicians | D6 | 47 |
| | | | | | | | RP7.S26 | Audit and provide feedback | D1 | 2 |
| RP7 | A14 | Third party provider (Previon) presented to all CHCs in order to obtain buy-in | RP7.P15 | Facilitation | D2 | 11 | RP7.S27 | Obtain formal commitments | D4 | 24 |
| | A15 | Culturally and linguistically tailored materials for interventions | RP7.P16 | Tailor strategies | D3 | 15 | RP7.S28 | Develop educational materials | D5 | 38 |

| | | | | | | | | | | |
|---|---|---|---|---|---|---|---|---|---|---|
| | | | | | | | RP7.S29 | Distribute educational materials | D5 | 40 |
| | | | | | | | RP7.S30 | Intervene with patient/ consumers to enhance uptake and adherence | D7 | 52 |
| | A16 | Centralize apporach to mailed FIT outreach using a thrid party vendor | RP7.P17 | Centralize technical assistance | D2 | 14 | RP7.S31 | Change service sites | D9 | 70 |
| | | | | | | | RP7.S32 | Change physical structures and equipment | D9 | 68 |
| | A17 | Dissemination of Usual Care Assessment | RP7.P18 | Conduct local needs assessment | D1 | 7 | RP7.S33 | Assess readiness and identify barriers and facilitators | D1 | 1 |
